# Morphological Evaluation of Vascular Foramina in Dry Adult Human Calcanei: A Foraminal Index-Based Study From Western India

**DOI:** 10.7759/cureus.112706

**Published:** 2026-07-15

**Authors:** Jahnavi Nath, Hetal Vaishnani, Bhoopesh Raja

**Affiliations:** 1 Department of Anatomy, Smt. B. K. Shah Medical Institute and Research Centre, Sumandeep Vidyapeeth (Deemed to be University), Vadodara, IND

**Keywords:** calcaneus, dry bone study, foraminal index, nutrient artery, surgical anatomy, tarsal bone anatomy, vascular foramen

## Abstract

Introduction and aim: The calcaneus is the largest tarsal bone and bears a major share of body weight during locomotion. Its vascular supply enters through small openings called vascular (nutrient) foramina, which lie on the medial, lateral, superior, and inferior surfaces. Knowledge of their number, location, and distribution is clinically relevant during calcaneal osteotomy, fracture fixation, and reconstructive procedures, as inadvertent injury to these vessels may precipitate delayed healing, non-union, or avascular necrosis. This study aimed to evaluate the morphology, number, location, and foraminal index (FI) of vascular foramina on the four anatomical surfaces of dry adult human calcanei from the Vadodara region.

Materials and methods: A descriptive, cross-sectional, observational study was conducted on 40 intact, non-pathological dry adult human calcanei from the bone bank of the Department of Anatomy, Smt. B. K. Shah Medical Institute and Research Centre, Vadodara. Specimens with macroscopic damage or deformities were excluded. The total length (TL) of each calcaneus was measured between the most prominent anterior and posterior points using a manual vernier caliper of 0.02 mm precision. The distance from the most posterior point to the largest vascular foramen on each of the four surfaces was recorded as the largest medial surface foramen (LMS), largest lateral surface foramen (LLS), largest superior surface foramen (LSS), and largest inferior surface foramen (LIS). The foraminal index for each surface was calculated as (distance/TL) × 100. Each measurement was taken twice by the same observer, and the mean value was used to minimize observer bias. Descriptive statistics (mean, standard deviation, range, and percentage) were computed using Microsoft Excel (Redmond, WA: Microsoft Corp.).

Results: The mean total length of the calcanei was 74.87 ± 4.82 mm (range: 63.06-86.50 mm). At least one vascular foramen was identified on every specimen. Foramina were most frequently observed on the lateral surface (36/40, 90.0%) and the medial surface (35/40, 87.5%), followed by the inferior surface (29/40, 72.5%) and least often on the superior surface (13/40, 32.5%). The mean foraminal index was 54.84 ± 12.65% on the medial surface, 41.67 ± 11.86% on the lateral surface, 35.65 ± 13.38% on the superior surface, and 56.30 ± 22.55% on the inferior surface. The majority of medial (27/35, 77.1%) and lateral (29/36, 80.6%) foramina were located in the middle third of the calcaneal length, while inferior foramina were more widely scattered between the middle and distal thirds.

Conclusion: Vascular foramina of the calcaneus exhibit considerable variability in number, location, and distribution. They are most consistent on the medial and lateral surfaces, where the bulk of the foramina cluster around the mid-calcaneal region. These observations may aid orthopedic surgeons in planning safe surgical corridors during calcaneal osteotomy, fracture fixation, and graft harvesting, thereby reducing the risk of vascular compromise and improving postoperative bone healing.

## Introduction

The calcaneus is the largest and strongest tarsal bone, forming the posterior framework of the hindfoot. It transmits body weight from the talus to the ground and serves as the lever arm for the Achilles tendon during plantar flexion and gait propulsion [1,2]. Its complex three-dimensional architecture, composed of dense cortical shell over a richly trabecular core, demands an equally elaborate vascular network for normal homeostasis, growth, and remodeling [1,3].

The vascular supply of the calcaneus is delivered by small branches of the posterior tibial, peroneal, and dorsalis pedis arteries that enter through nutrient (vascular) foramina distributed unevenly over its four anatomical surfaces - medial, lateral, superior, and inferior [3-5]. These foramina are not arbitrarily placed; their location and direction are determined by the pattern of bone growth and by the regional functional demands acting on the bone during development [6]. Once established, the size and orientation of the dominant nutrient foramen remain important anatomical determinants of intramedullary blood flow throughout life.

From a clinical standpoint, knowledge of the topography of these foramina is far from academic. Calcaneal fractures account for approximately 60% of all tarsal injuries, and operative management, including open reduction with internal fixation, lateral extensile approaches, and calcaneal osteotomies, exposes the principal nutrient vessels to inadvertent injury [3,4,7]. Disruption of these vessels has been implicated in delayed union, non-union, and post-traumatic avascular necrosis of the posterior tuberosity [4,5]. Vascular foramina may also be misinterpreted as pathological lesions on radiographs and computed tomography, leading to unnecessary investigations [3].

Although the topography of nutrient foramina in long bones has been studied extensively [5,6,8], comparatively few studies have characterized them in the tarsal bones [3,4,9]. Recent morphometric work on the lower-limb skeleton has further emphasized that the number, position, and direction of nutrient foramina vary across populations and that this variability is biomechanically and surgically meaningful [9,10]. To the best of the present authors' knowledge, dedicated dry-bone data from the Vadodara region of western Gujarat are limited. The present study was therefore undertaken to document the morphology, number, location, and foraminal index of vascular foramina on the four anatomical surfaces of 40 dry adult human calcanei from this region.

## Materials and methods

Study design and setting

A descriptive, cross-sectional, observational study was conducted in the Department of Anatomy, Smt. B. K. Shah Medical Institute and Research Centre, Sumandeep Vidyapeeth (Deemed to be University), Vadodara, Gujarat, India. The study protocol was reviewed and approved by the Sumandeep Vidyapeeth Institutional Ethics Committee (SVIEC) prior to commencement of data collection. As the study used only fully skeletonized, dry adult calcanei from the institutional bone collection and did not involve human participants, animals, or identifiable personal data, written informed consent was not applicable. Data collection was carried out over a period of approximately three months, from March 5, 2026, to June 10, 2026.

Specimens

A total of 40 dry, fully ossified, adult human calcanei of unknown sex were retrieved from the osteology repository of the Department of Anatomy. Specimens were assigned arbitrary identifiers (A1-A50) for traceability.

Inclusion and exclusion criteria

Intact, fully ossified, adult dry calcanei free of gross damage or pathological alteration were included. Calcanei with fractures, erosions, surgical artifacts, marked deformity, or extensive surface erosion that could obscure foraminal identification were excluded.

Instruments

All linear measurements were obtained with a standard manual vernier caliper of 0.02 mm precision. A hand-held magnifying lens (×3) and a probing needle of 0.4 mm gauge were used to confirm patency of each candidate foramen and to distinguish true vascular foramina from non-vascular pits and erosions. A high-resolution digital camera (Canon EOS 1500D 24.1 MP DSLR with an 18-55 mm kit lens; Tokyo, Japan: Canon Inc.) was used for photographic documentation.

Identification of vascular foramina

A vascular foramen was operationally defined as a clearly demarcated, patent opening in the bone surface through which a fine-gauge probing needle could be passed a measurable distance into the medullary cavity. Each calcaneus was systematically inspected on its following four anatomical surfaces: medial, lateral, superior, and inferior. When several foramina were present on a single surface, the largest foramen was identified by direct visual inspection aided by a magnifying lens and used as the reference for distance measurements on that surface.

Measurements

**Figure 1:**
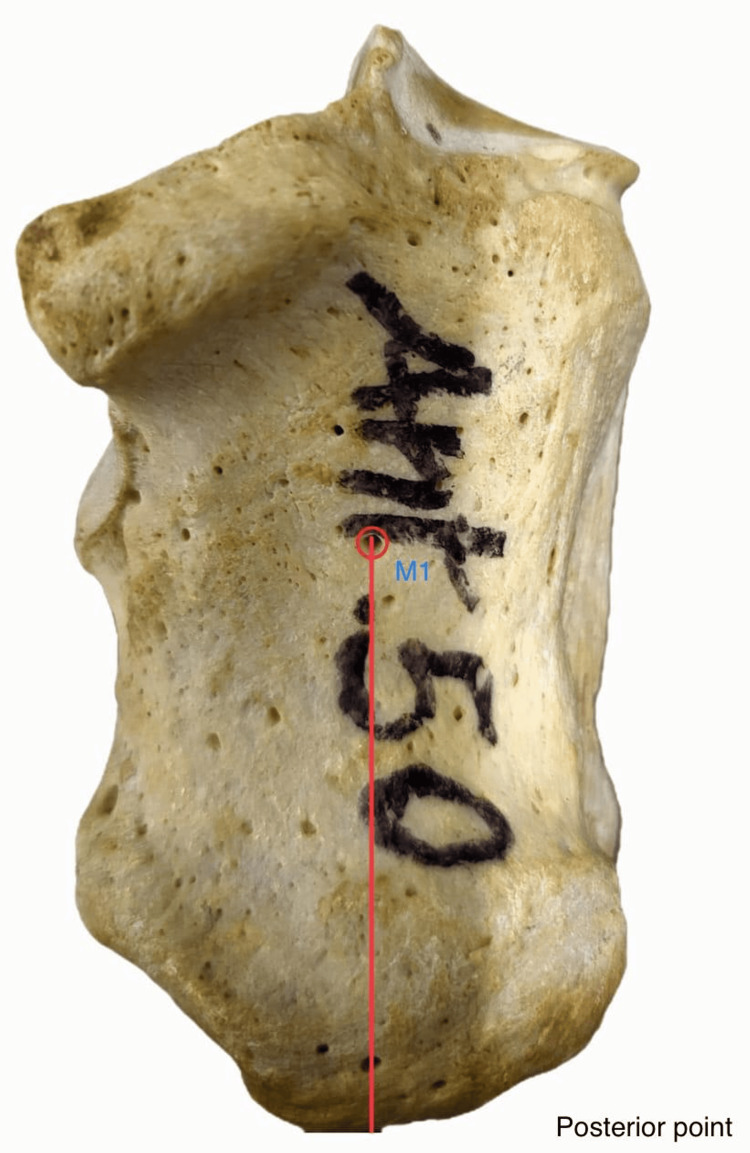
Measurement of distance from the posterior point to the largest medial vascular foramen (LMS).

**Figure 2:**
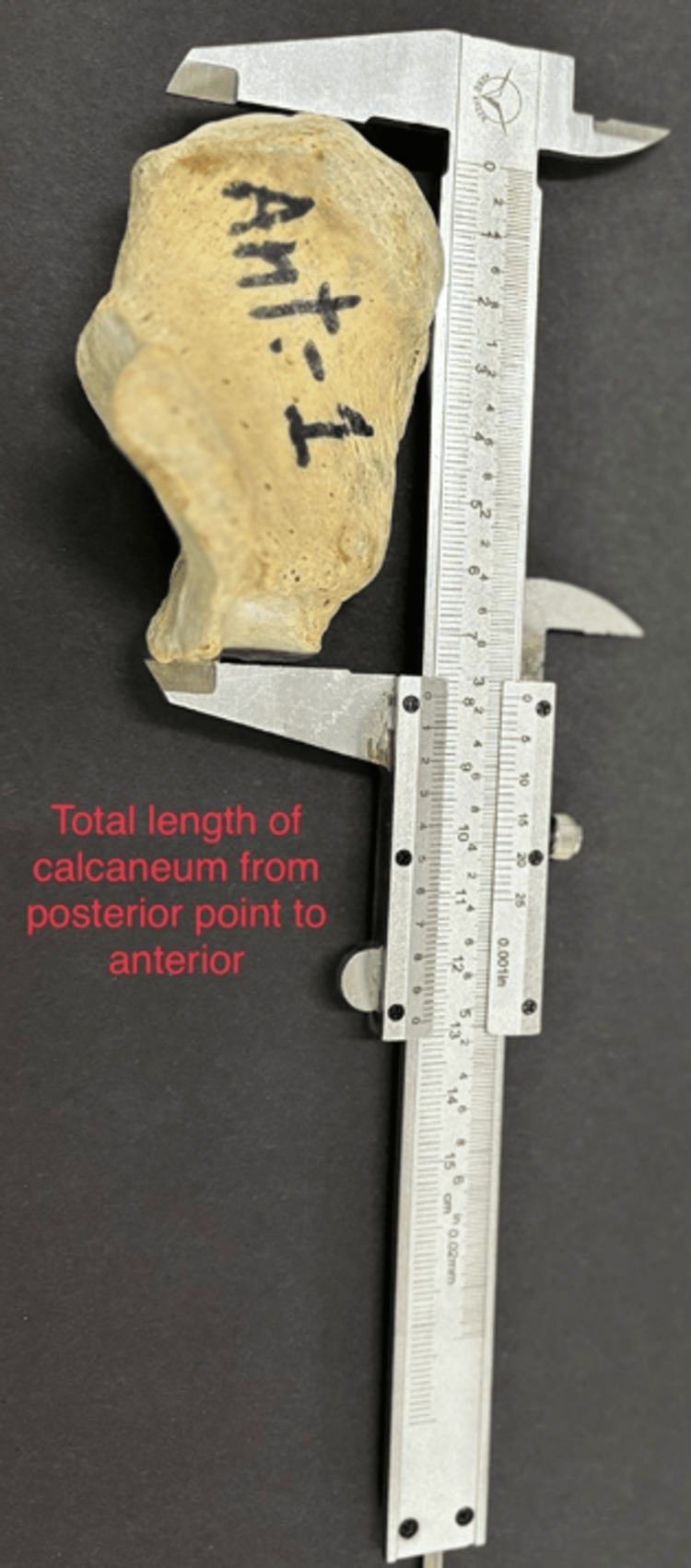
Measurement of total calcaneal length (TL) using a standard manual vernier caliper.

**Figure 3:**
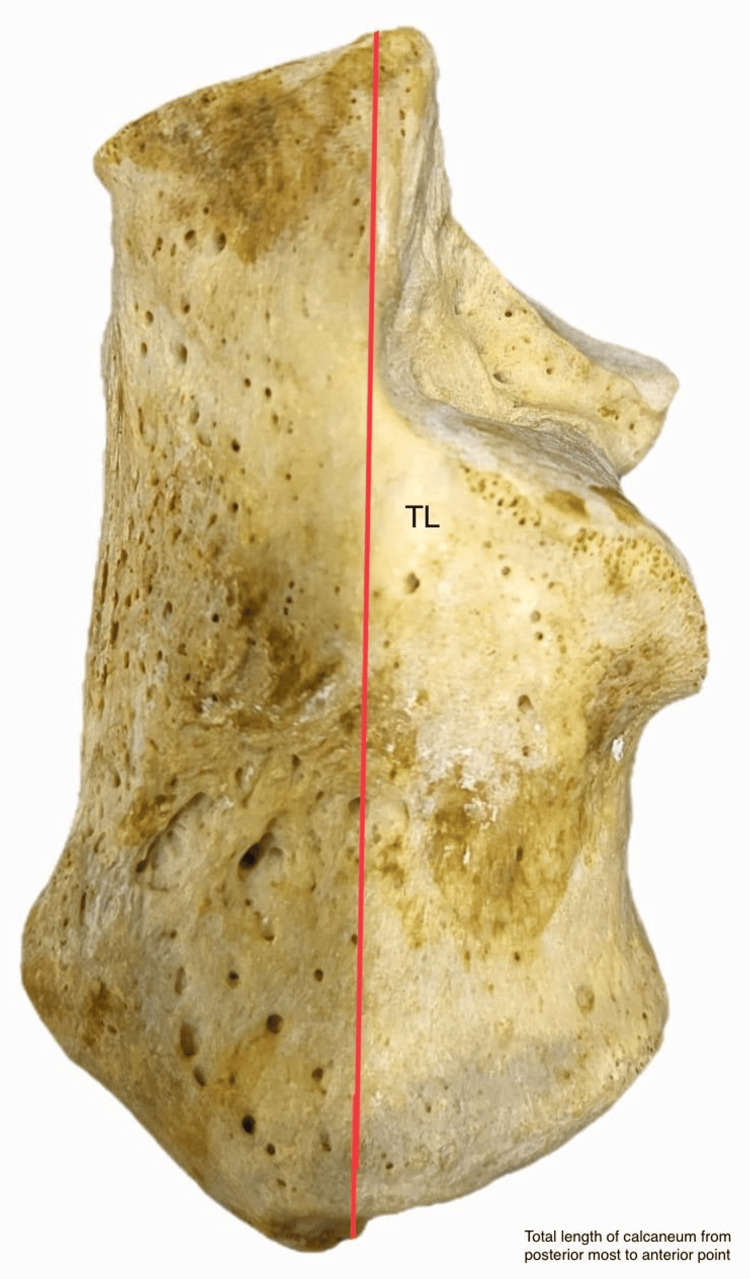
Measurement of total calcaneal length (TL).

**Figure 4:**
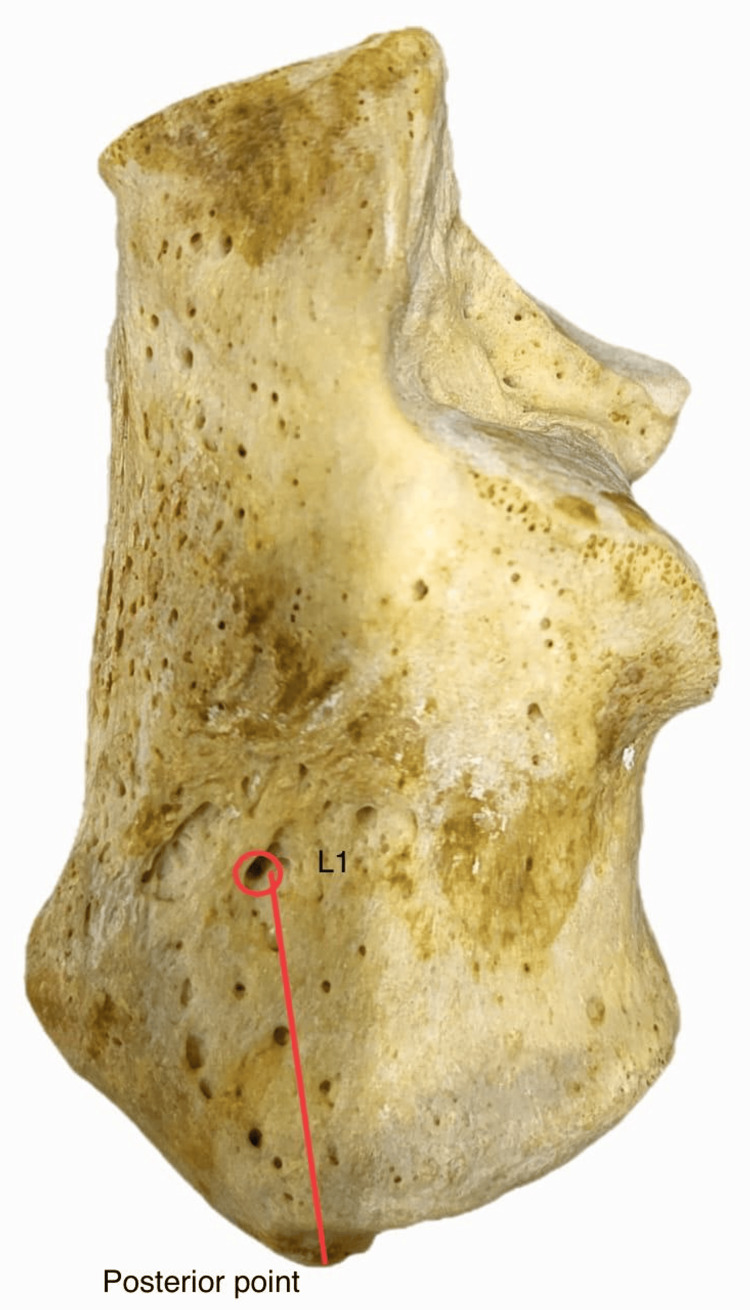
Measurement of distance from the posterior point to the largest lateral surface foramen (LLS).

The following five linear parameters were recorded for each calcaneus and expressed in millimeters. Total length (TL) was defined as the maximum sagittal length measured from the most prominent anterior point of the calcaneus to the most prominent point of the posterior calcaneal tuberosity, with the calcaneus positioned in standard anatomical orientation. The distances from the most prominent point of the posterior calcaneal tuberosity to the center of the largest medial surface foramen (LMS), the largest lateral surface foramen (LLS), the largest superior surface foramen (LSS), and the largest inferior surface foramen (LIS) were measured in a straight line using a manual vernier caliper (Figures 1-4).

Each measurement was repeated twice by the same observer with the caliper jaws reapplied, and the arithmetic mean was used to minimize intraobserver variability. When no foramen was identified on a given surface, that surface was recorded as absent and excluded from the corresponding analysis. The foraminal index (FI) for each surface was calculated from the standard formula.

$FI = \left( \frac{\text{distance from posterior point to largest foramen}}{\text{total length}} \right) \times 100$

Thus, FI1, FI2, FI3, and FI4 represent the foraminal indices of the medial, lateral, superior, and inferior surfaces, respectively [6,9]. The position of each foramen along the calcaneal length was further categorized using Hughes' classical scheme as follows: proximal third (FI < 33.3%), middle third (FI = 33.3-66.6%), and distal third (FI > 66.6%) [6].

Statistical analysis

All measurements were tabulated in Microsoft Excel (Redmond, WA: Microsoft Corp.). Descriptive statistics, mean, standard deviation (SD), minimum, maximum, and proportions, were computed for every parameter. Categorical data such as surface-wise presence of foramina and zonal distribution were summarized as counts and percentages. Because the study was descriptive in design, no inferential hypothesis testing was undertaken; p-value computations were therefore not applicable. A p-value of less than 0.05 would have been considered statistically significant had any inferential test been required.

## Results

A total of 40 dry adult human calcanei were analyzed. At least one vascular foramen was identified in each specimen, confirming that the calcaneus is consistently vascularized via surface foramina. The mean total length of the calcanei was 74.87 ± 4.82 mm, with a range of 63.06-86.50 mm.

Sample composition

The sample composition of the studied calcanei is summarized in Table 1. A total of 40 dry adult human calcanei were included in the study, comprising 23 (57.5%) right-sided and 17 (42.5%) left-sided specimens. Side identification was recorded during cataloging in the bone section of the department and verified before morphometric measurements were performed. All specimens were examined irrespective of side to ensure an adequate representation of the available sample for morphological and morphometric analysis.

**Table 1:** Sample composition of the studied calcanei. Side identification was recorded at the time of cataloging in the bone bank.

Parameters	n	%
Total calcanei studied	40	100.0
Right-sided calcanei	23	57.5
Left-sided calcanei	17	42.5

Morphometric measurements

The mean and standard deviation of the morphometric measurements (in millimeters) are presented in Table 2. Of note, the denominators differ across surfaces because, on calcanei lacking a foramen on a given surface, the corresponding distance was not measurable.

**Table 2:** Mean and standard deviation of morphometric measurements. TL was measurable in all 40 specimens. n: number of calcanei in which the corresponding surface bore at least one identifiable vascular foramen; LMS: largest medial surface foramen; LLS: largest lateral surface foramen; LSS: largest superior surface foramen; LIS: largest inferior surface foramen; TL: total length

Parameters	n	Mean (mm)	SD (mm)	Range (mm)
TL	40	74.87	4.82	63.06-86.50
LMS	35	40.74	8.92	18.60-54.60
LLS	36	31.14	9.59	19.58-62.60
LSS	13	27.45	12.0	19.80-64.30
LIS	29	42.51	17.09	17.32-65.24

Surface-wise distribution of vascular foramina

**Figure 5:**
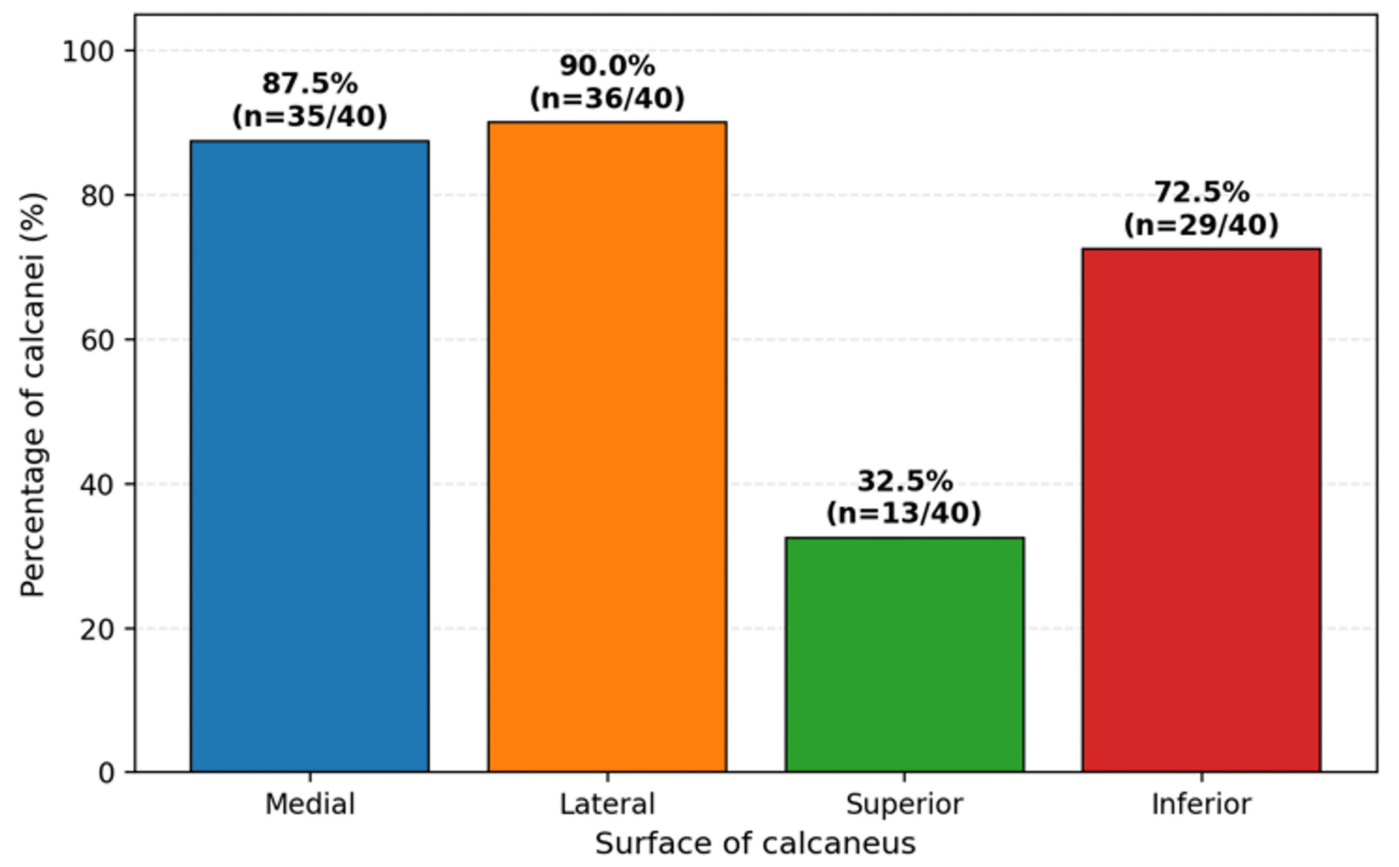
Surface-wise distribution of vascular foramina in 40 dry calcanei. The lateral and medial surfaces are most consistently vascularized.

Among the examined calcanei, vascular foramina were absent on the medial surface in five specimens, on the lateral surface in four specimens, on the superior surface in 27 specimens, and on the inferior surface in 11 specimens. The lateral surface most frequently bore at least one vascular foramen (36 of 40, 90.0%), followed closely by the medial surface (35 of 40, 87.5%) and the inferior surface (29 of 40, 72.5%). The superior surface was the least frequent location, with foramina present in only 13 of 40 specimens (32.5%) (Figure 5, Table 3). When the per-bone count was tallied, only one specimen (2.5%) carried a foramen on a single surface; 10 (25.0%) had foramina on two surfaces, 24 (60.0%) on three surfaces, and five (12.5%) on all four surfaces, giving a mean of 2.83 ± 0.68 surfaces with foramina per calcaneus (Figure 5, Table 3).

**Table 3:** Surface-wise presence of vascular foramina (n = 40 calcanei).

Surface	Calcanei with foramen (n)	Presence (%)
Lateral	36	90.0
Medial	35	87.5
Inferior	29	72.5
Superior	13	32.5

Foraminal index

**Figure 6:**
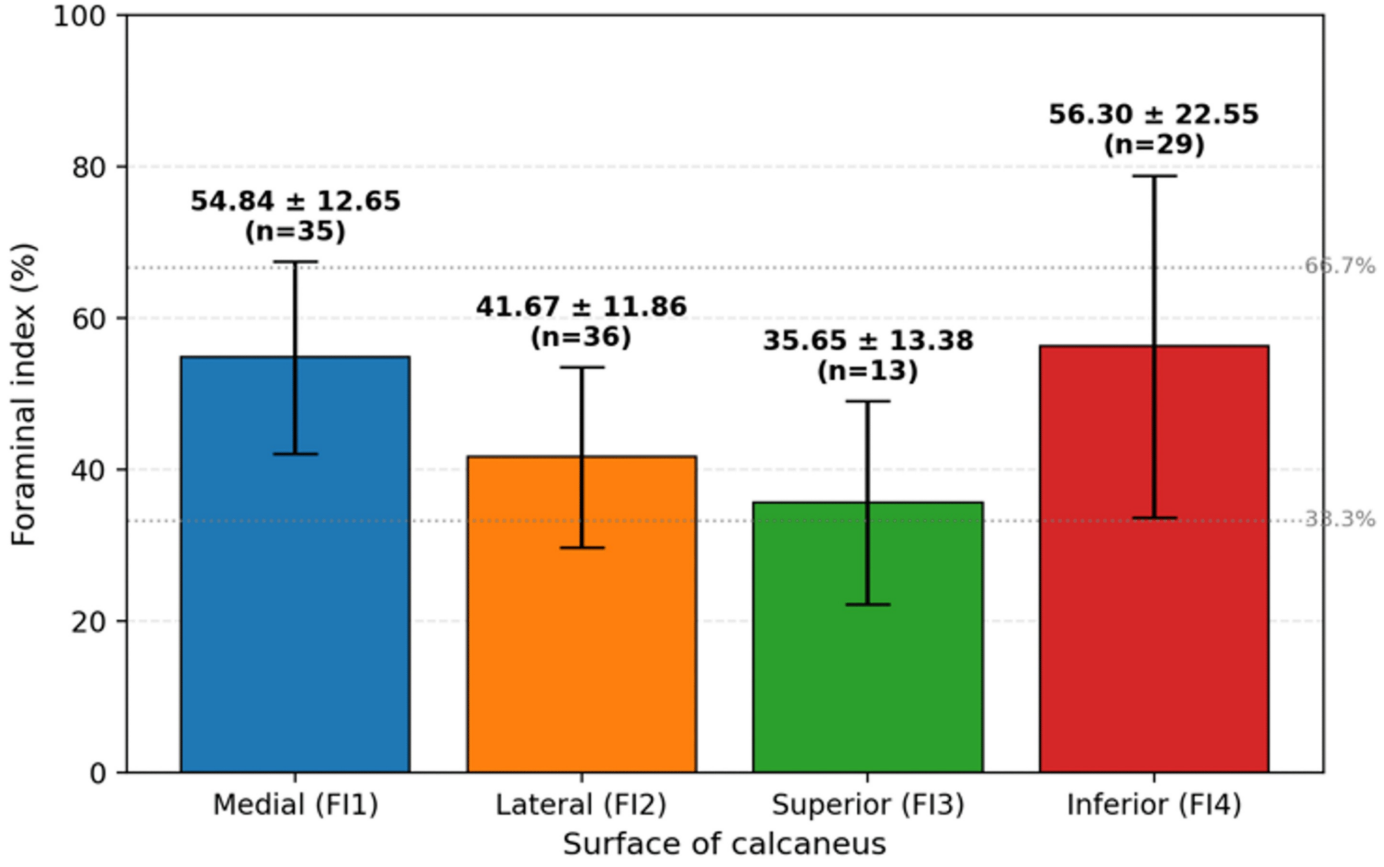
Mean foraminal index (±SD) of the largest vascular foramen on each of the four surfaces of the calcaneus. The dotted lines mark the boundaries between the proximal, middle, and distal thirds (33.3% and 66.7%).

The foraminal index, expressing the location of the largest foramen as a percentage of total calcaneal length, varied widely across surfaces (Figure 6, Table 4). The mean FI was highest on the inferior surface (56.30 ± 22.55%) and the medial surface (54.84 ± 12.65%), indicating that the dominant foramen on each of these surfaces tends to be situated slightly distal to the geometric midpoint of the bone. The lateral-surface FI averaged 41.67 ± 11.86%, placing its dominant foramen within the proximal half of the middle third. The superior surface, when a foramen was present, showed the lowest mean FI of 35.65 ± 13.38%, i.e., closer to the proximal end (Figure 6, Table 4).

**Table 4:** Mean and standard deviation of the foraminal index (FI) for each surface.

Foraminal index	n	Mean (%)	SD (%)	Range (%)
FI1 (medial)	35	54.84	12.65	25.20-83.70
FI2 (lateral)	36	41.67	11.86	26.60-80.90
FI3 (superior)	13	35.65	13.38	25.60-74.30
FI4 (inferior)	29	56.30	22.55	24.31-85.60

Zonal distribution according to foraminal index

**Figure 7:**
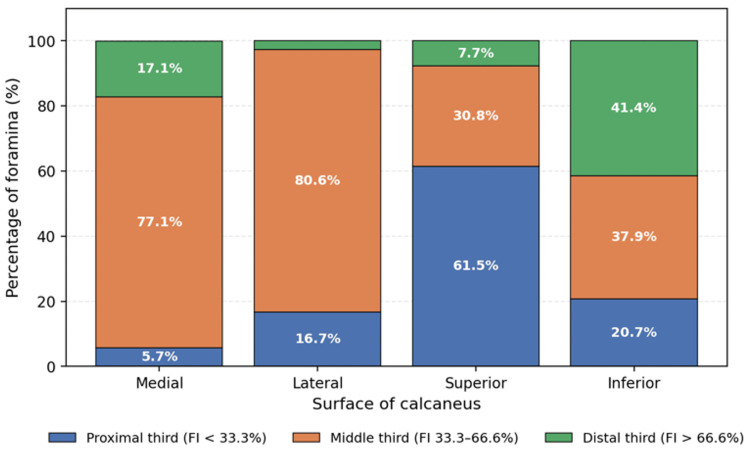
Zonal distribution of the largest vascular foramen on each surface, based on the foraminal index. Medial and lateral surfaces show a strong predilection for the middle third of the calcaneus.

When the foraminal index was used to allocate each foramen to one of three equal calcaneal zones (proximal, middle, or distal third), distinct patterns emerged across the four surfaces. Both the medial (27/35, 77.1%) and the lateral (29/36, 80.6%) surfaces showed a strong predilection for the middle third of the bone, indicating that the principal nutrient channels on the sides of the calcaneus enter very close to the geometric center of the bone. By contrast, the superior surface, when vascularized at all, had the majority of its foramina (8/13, 61.5%) sited in the proximal third, near the posterior tuberosity. The inferior surface displayed the most dispersed pattern, with foramina distributed almost equally between the middle (11/29, 37.9%) and the distal (12/29, 41.4%) thirds. Few foramina (0%-20.7%) were located in the proximal third of the medial, lateral, or inferior surfaces (Figure 7, Table 5).

**Table 5:** Zonal distribution of foramina according to the foraminal index (Hughes' classification). Proximal third: FI < 33.3%, middle third: FI 33.3-66.6%, distal third: FI > 66.6%.

Surface	n	Proximal third (%)	Middle third (%)	Distal third (%)
Medial	35	5.7	77.1	17.1
Lateral	36	16.7	80.6	2.8
Superior	13	61.5	30.8	7.7
Inferior	29	20.7	37.9	41.4

## Discussion

The present study characterized the morphology and topography of vascular foramina on 40 dry adult human calcanei from the Vadodara region. The principal findings were that vascular foramina were universally present in the calcaneus, that the lateral and medial surfaces were the most consistently vascularized, and that the dominant foramina on the medial and lateral surfaces clustered in the middle third of the bone, whereas those on the inferior surface were more widely scattered. These observations have direct implications for orthopedic surgery and reinforce the long-standing anatomical view that the calcaneus is a vascularly resilient bone, supplied through multiple cortical entry points rather than relying on a single dominant nutrient channel [3-5].

Total length of the calcaneus

The mean total length of the calcanei in this series (74.87 ± 4.82 mm) is consistent with values reported in earlier Indian morphometric studies. Prasad and Rajasekhar reported comparable calcaneal dimensions in an Indian dry-bone sample [3], while Anbumani et al. documented similar calcaneal morphometry in a South Indian population [7]. The narrow standard deviation observed in the present series (about 6.4% of the mean) likely reflects the relatively homogeneous regional origin of the specimens and supports the use of total length as a stable denominator for the foraminal index. By contrast, morphometric work from other populations has emphasized that the dimensions and nutrient-foramen topography of skeletal elements vary with stature and body composition [8,9].

Number and surface-wise distribution of vascular foramina

Every calcaneus in the present series carried at least one identifiable vascular foramen, and the mean number of surfaces bearing foramina per bone was 2.83 ± 0.68. This is broadly in line with previous reports, which describe the calcaneus as one of the most consistently vascularized tarsal bones, typically supplied by three to five nutrient foramina distributed over its surfaces [3-5]. The strong preponderance of foramina on the lateral (36/40, 90.0%) and medial (35/40, 87.5%) surfaces accords with the classical observation that the principal extraosseous arterial supply of the calcaneus, branches from the posterior tibial and peroneal arteries, approaches the bone along its medial and lateral aspects, where soft tissues are loosest and vascular access is greatest [3,11].

The relatively low frequency of foramina on the superior surface (13/40, 32.5%) was an expected finding. The superior surface of the calcaneus is dominated by the posterior, middle, and anterior talar articular facets, which are covered by hyaline cartilage in life and offer little exposed cortical bone through which a foramen might pass [1,12]. Prasad and Rajasekhar and Ülkir and Gündoğdu likewise reported the superior surface as the least foramen-bearing zone [3,4]. The inferior surface, in contrast, has a much larger non-articular area, and the present finding of foramina on 29/40 (72.5%) of inferior surfaces is consistent with the literature [4,5].

Foraminal index and zonal distribution

The foraminal index provides a length-normalized, dimensionless descriptor of foraminal position that allows comparison between bones of different sizes and across studies. In the present series, the mean FI was highest on the inferior (56.30 ± 22.55%) and medial (54.84 ± 12.65%) surfaces and lowest on the superior surface (35.65 ± 13.38%). Application of Hughes' classical tripartite scheme disclosed that the majority of medial (27/35, 77.1%) and lateral (29/36, 80.6%) foramina occupied the middle third of the calcaneal length [6]. This zone corresponds anatomically to the region inferior to the sustentaculum tali medially and just posterior to the peroneal trochlea laterally, and it is the area where the principal nutrient vessels are known to enter the bone [11,13]. The dispersed pattern on the inferior surface (middle third 11/29, 37.9%; distal third 12/29, 41.4%) mirrors the gross anatomy of the plantar aspect, where multiple smaller branches enter the bone rather than a single dominant channel.

A practical surgical implication follows from these findings. The lateral extensile (Letournel-type) approach to the calcaneus, widely used in operative fixation of intra-articular calcaneal fractures, raises a full-thickness flap whose vascular base lies very close to the middle third of the lateral surface, precisely where the present study located 29/36 (80.6%) of the lateral foramina. The high incidence of wound-edge necrosis described with this approach has been mechanistically linked to disruption of these foramina [4,11,14]. Similarly, medial slide calcaneal osteotomies and posterior tuberosity osteotomies pass through, or near, the middle and distal thirds of the bone, where medial and inferior foramina are concentrated. Awareness of this topography may help the surgeon to plan flap design and osteotomy lines that preserve, rather than transect, the dominant nutrient channels.

Comparison with previous studies

The present results are broadly concordant with previous Indian and international morphometric studies on calcaneal vascular foramina [3,4]. Prasad and Rajasekhar found a predominance of foramina on the medial and lateral surfaces of the calcaneus [3]. Ülkir and Gündoğdu similarly highlighted the clinical relevance of the lateral and medial foramina for surgical approaches to the calcaneus [4]. Mysorekar's foundational work on long bones established that the direction and concentration of nutrient foramina reflect both bone-growth patterns and regional functional demands, a generalization that the present calcaneal data continue to support [6]. More recently, Zahra et al. emphasized the importance of population-specific data on nutrient foramina for surgical planning, building on the classical framework established by Hughes [9,10]. The present study contributes to the existing body of literature by providing regional data from Western India, where dedicated dry-bone analyses of the calcaneus are scarce. Additional studies by Gümüşburun et al., Vani et al., Bridgeman and Brookes, and Prasarn et al. further support the importance of nutrient foramina patterns and vascularization in skeletal anatomy and surgical planning [15-18].

Strengths and limitations

The principal strengths of the present study are the use of an objective foraminal index, the application of Hughes' standardized zonal classification, the dual measurement of every parameter to reduce observer bias, and the report of results in a format that can be directly compared with future studies. The study is, however, subject to several limitations. First, the sample size of 40 dry calcanei, although comparable to several published series in this field, is modest and may limit the statistical precision of the proportion estimates and the generalizability to other Indian regions. Second, the sex, age, and stature of the bones were unknown because the specimens were drawn from an institutional bone bank; this precluded sex-wise and age-related sub-analyses. Third, the study examined only the largest foramen on each surface; smaller accessory foramina, although noted, were not individually quantified. Fourth, the patency of each foramen was confirmed only with a fine probing needle and could not be cross-checked by perfusion or radiographic techniques on the dry specimens. Future work might address these limitations through a larger, sex-stratified series with concurrent imaging or vascular casting, and through bilateral comparisons in matched specimens.

## Conclusions

Vascular foramina of the dry adult calcaneus exhibit substantial inter-bone variability in number, location, and distribution. In the present series of 40 specimens from the Vadodara region, foramina were most consistently identified on the lateral (36/40, 90.0%) and medial (35/40, 87.5%) surfaces, were less frequent on the inferior surface (29/40, 72.5%), and were least frequent on the superior surface (13/40, 32.5%). The dominant foramina on the medial and lateral surfaces tended to cluster within the middle third of the calcaneal length, as defined by the foraminal index, whereas inferior foramina were more widely scattered between the middle and distal thirds.

These findings provide regional reference data that may inform safer surgical planning for calcaneal osteotomy, fracture fixation, and reconstructive procedures, particularly when the lateral extensile and medial slide approaches are contemplated. By avoiding the foramen-rich middle third of the medial and lateral surfaces wherever clinically feasible, the operating surgeon may help to preserve the principal nutrient supply of the bone and thereby reduce the risk of delayed union, non-union, and post-operative wound-edge necrosis. Larger, sex-stratified, and imaging-correlated studies are now needed to refine these reference values for diverse Indian and global populations.
